# Ecotoxicological Risk Assessment of Actellic 50 EC Insecticide on Non-Target Organisms in Parallel with the Application of Standardized Tests

**DOI:** 10.3390/toxics10120745

**Published:** 2022-11-30

**Authors:** Alina Paunescu, Cristina Maria Ponepal, Lucica Tofan, Gheorghita Brinzea, Monica Marilena Tantu, Cristina Florina Mihaescu, Oana Alexandra Draghiceanu, Dan Razvan Popoviciu, Marius Mirodon Fagaras, Daniela Vasile, Liliana Cristina Soare

**Affiliations:** 1 Natural Science Department, Faculty of Sciences, Physical Education and Informatics, University of Pitesti, 110040 Pitesti, Romania; 2 Faculty of Natural and Agricultural Sciences, Ovidius University of Constanta, 900470 Constanta, Romania

**Keywords:** Actellic 50 EC, wheat, ferns, fish, marsh frog tadpoles, Phytotoxkit microbiotest

## Abstract

This paper contributes to the ecotoxicological risk assessment of the Actellic 50 EC insecticide (with 50% pirimiphos-methyl as the active substance) tested on non-target organisms. The insecticide concentrations tested were the same for all organisms (0.1, 0.01, and 0.001 mg L^−1^ of Actellic 50 EC), with an exposure of 3–5–21 days for plants and 4–5–14 days for animals. The non-target organisms tested were both plants (wheat and two ferns) and animals (the Prussian carp and marsh frog tadpoles). The tested insecticide significantly inhibited the growth of roots in wheat, a result that was also confirmed by a microbiotest application (62% root growth inhibition in sorghum and 100% germination inhibition in white mustard and garden cress). In ferns, even for the lowest concentration, the percentage of germinated spores was inhibited by 40% for *Asplenium scolopendrium*. The recorded toxicological effects of Actellic 50 EC upon the Prussian carp included a decrease in the respiratory rate and oxygen consumption, an increase in the number of erythrocytes and leukocytes, and an increase in blood glucose levels. The highest concentration (0.1 mg L^−1^ of Actellic 50 EC) caused a 50% decrease in the survival rate of marsh frog tadpoles after 5 days of exposure, negatively affecting body volume and length. Given the high degree of toxicity of the insecticide Actellic 50 EC, we recommend continuing investigations on non-target species, including both plants and animals, as the sub-chronic effects are quite little known in the scientific literature.

## 1. Introduction

The intensive development of agriculture to meet the needs of the global economy to provide enough food for an exponentially growing population is leading to new pesticides to ensure plant protection and higher crop yields. Among pesticides, organophosphates have been introduced because of their lower persistence in the environment compared to organochlorines [1]. In addition, they do not accumulate in tissues and have a lower risk of transfer along food chains [1]. Although they are less persistent in the environment, they can have harmful effects not only on the pests for which they are prescribed, but also on non-target species, including fish, plants, soil microorganisms, and beneficial insects, leading to significant biodiversity losses and implicitly increasing the vulnerability of ecosystems to changes in the environment [2]. A summary of the cumulative dietary risk assessments of pesticides during 2012–2017 in Denmark indicated an exposure of 0.023 μg-kg^−1^ body weight/day to pirimiphos-methyl in adults aged 15–75 years and a hazard quotient (HQ) of 0.0058, and moreover, for children aged 4–6 years, an HQ greater than 0.01 [3]. 

The toxicity tests for the approval of Actellic 50 EC as a commercial product were carried out mainly on laboratory animals (rats, mice, and dogs) and were related to the use of the insecticide for phytosanitary protection only in indoor storage spaces [4]. 

In a recent posting, one of the Actellic 50 EC manufacturers provided information [5] on the ecological and toxicological risks when the insecticide is not handled according to the data sheet and label instructions: 0.64 mg L^−1^ LC50 (96 hours) in rainbow trout; 0.21 µg L^−1^ LC50 (48 hours) in *Daphnia*; and 1.0 mg L^−1^ EC50 in algae. Biodegradation is reported to take between 3.5 and 30 days (DT50-soil), but mutagenic and carcinogenic effects are not known by this producer.

The active ingredient of Actellic 50 EC is pirimiphos-methyl [6]. It is slightly volatile, having a low water solubility (10 mg L^−1^ in unbuffered water at 20 °C) and a mean hydrolysis time (DT_50_) of 75 days at a pH of 9 and 25 °C [7]. Following hydrolysis, two compounds result: O-2-diethylamino-6-methylpyrimidin-4-yl O-methyl phosphorothioate (<10% at a pH of 4–7; 13% at a pH of 9) and 2-diethylamino-6-methylpyrimidin-4-ol (>90% at a pH of 4–5; 7.7–12% at a pH of 7–9). The photolysis half-life (DT50) is 0.46 h at a pH of 5 and 25 °C, and 0.47 h at a pH of 7 and 25 °C [7]. It is classified by the WHO as belonging to toxicity class III [8]. Even though the manufacturers of this insecticide recommend its use only indoors for phytosanitary treatments, this product has been used to control mosquito larvae and can thus reach various bodies of water [9]. Thus, in India, studies have been conducted on the use of this insecticide as a larvicide in clean and polluted waters against anophelines and culicines, but it could be toxic for the larval stages of fish in aquatic ecosystems at concentrations higher than 0.25 ppm [10]. The repeated application of treatments for mosquito larva control can result in, among other things, subcellular damage to fish species (non-target organisms) [9]. Fish mortality due to organophosphorus pesticide poisoning has been reported in the literature even at low concentrations (ng–μgmL^−1^), because these compounds are acetylcholinesterase inhibitors [11,12]. There is an inverse relationship between the water solubility of insecticides and their accumulation in fish tissues and organs [13]. Their effects on aquatic organisms depend on the compound, exposure time, water quality, and species [14]. Due to the fact that fish absorb, metabolize, and excrete organophosphorus compounds rapidly, chronic exposure is not considered a real problem [15].

The LC_50_ 48-hour values for the commercial product Actellic 50 in the adults and juveniles of *Oreochromis mossambicus* at 28–29 °C under static conditions were reported to be 1.3804 and 2.1928 mg L^−1^, respectively [16]; the authors reported spinal deformities at a concentration of 0.4 mg L^−1^of Actellic 50, as well as excess mucus around the eyes. 

*Carassius gibelio* is an invasive species that is becoming dominant in Europe. The Prussian carp was chosen as the bioindicator species in this study, since it is a common species in Romanian rivers, ponds, and lakes; easily available and maintained under laboratory conditions; and highly resistant to adverse environmental effects and the presence of pollutants. 

Amphibians are more sensitive to the action of xenobiotics in the environment than other vertebrates, because they live in both aquatic and terrestrial environment and have a thin and highly permeable skin through which they exchange gases, water, and electrolytes with the environment [17]. Much of the amphibian life cycle occurs in ponds, streams, and temporary pools that are often associated with agricultural areas receiving pesticide applications [18,19]. Agricultural pesticides may contribute to the decline in amphibian populations [20], and it is therefore important to investigate the effect of pesticides. Taking into account all these factors, they can be considered good indicators for environmental quality.

Besides amphibian vertebrates and fish, the study also followed the effects of the spread of this insecticide on some plants (ferns, wheat, and other crop plants) that exist in areas at risk of contamination by improper use or storage of this insecticide.

We wanted to demonstrate the variability of the toxicity of the contaminated soils to plants, as well as the effectiveness and usefulness of the Phytotoxkit microbiotest as a practical and reliable tool [21] for assessing the risk of the Actellic 50 insecticide in the event of its spread in soil and surface waters.

The purpose of this research was to highlight the effects induced by Actellic 50 EC in exposed non-target organisms by evaluating:

- The germination and growth of seedlings, their biomass, and their assimilation of pigments for *Lepidium sativum*, *Sinapis alba*, and *Sorghum saccharatum*;
- The plant growth and biomass of *Triticum aestivum*;
- The germination of spores and the differentiation of gametophytes for *Athyrium filix-femina* and *Asplenium scolopendrium*;
- The survival rate, behavioral changes, respiratory rate, oxygen consumption, number of figurative elements, and blood glucose of *Carassius gibelio*;
- The successful hatching of *Pelophylax ridibundus* tadpoles, their survival rate, and changes in the morphology of the body.

## 2. Materials and Methods

### 2.1. Experiments on Plants 

The toxicity tests focused on the effects on the germination process and the early development of vegetative and reproductive organs for ferns, wheat, sorghum, white mustard, and garden cress. The last three plants are part of a standardized microbioassay commercially called Phytotoxkit.

#### 2.1.1. Application of the Phytotoxkit Microbiotest 

The Phytotoxkit is a 3-day bioassay [22] that is based on the seed germination and early root growth of 3 higher plant species after exposure to soils contaminated with Actellic 50 EC. The seeds of 2 dicotyledonous species (*Lepidium sativum* (garden cress) and *Sinapis alba* (white mustard)) and a monocotyledon species (*Sorghum saccharatum* (sorghum)) were exposed in triplicate to evaluate the effects of contaminated soil (72 h in darkness at 25 °C ± 2 °C) and control soil (reference or standard soils can be used as negative controls) [23,24]. Commercially available seeds, with a shelf life longer than one year, allow for the use of this test at any time of the year. 

The soil was contaminated with Actellic 50 EC at the same concentrations/variants of pirimiphos-methyl used for the other plants tested, i.e., A0.1 = 0.1 mg L^−1^, A0.01 = 0.01 mg L^−1^, and A0.001 = 0.001 mg L^−1^, with 3–5 days of exposure. After 3 days of exposure, the germination percentage and the degree of root growth inhibition were determined. 

After 5 days of exposure, the inhibition of shoot and leaf growth and the variation in dry biomass and pigments compared to the control group were determined. 

Plant root, shoot, and leaf length were determined by photographing plant batches with a 13 MP digital camera and analyzing the images using ImageJ software [25]. 

The plant biomass at the end of the experiment was determined by oven drying each batch of sorghum plants at 105 °C for 5 h and weighing the dry residue. The average dry biomass per plant was determined by considering the number of plants taken for analysis from each batch.

The concentration of foliar pigments was determined by grinding 0.1 g of leaf tissue in 10 mL of acetone solution (80%). The extract was filtered and its spectrophotometric absorbance was read at 470, 647, and 663 nm. The chlorophyll *a*, chlorophyll *b*, and total carotenoid concentrations were determined using specific equations [26].

#### 2.1.2. Application of the *Triticum* Test 

This test is commonly used to assess the toxicity of pesticides, heavy metals, drugs, nanomaterials, etc. [27], with wheat being one of the species used in the existing plant-testing guidelines [28,29]. Seeds of *Triticum aestivum* (Trivale variety) were provided from the Pitești Agricultural Research and Development Station, Albota. Seed sterilization (5 min in 75% ethanol) [30] was followed by hydration in distilled water (1 h) and then immersion in Actellic 50 EC solution (12 and 24 h) (Table 1). Distilled water was used for the control. The seeds were then placed on filter paper in Petri dishes and stored in the growth chamber; the root and stem length measurements were made 5 days after the exposure. After the fresh weight was determined, the plant material was placed in the oven at 80 °C to determine the dry weight. Ten seeds were used for each variant, and the experiment had 3 replications. 

#### 2.1.3. Contamination Tests on Fern Spores 

Fern spores and the gametophytes formed following their germination exhibit a number of characteristics suitable for ecotoxicological research [31,32], including germination on low-cost culture media, gametophyte sensitivity, and results that are relevant to eukaryotic organisms. We used spores from two fern species: *Athyrium filix-femina* and *Asplenium scolopendrium*. Spores were provided by the Romanian Pteridological Association. The spores were grown on the surface of liquid culture media (Knop solution with/without Actellic 50 EC) (Table 1). The culture dishes, covered and sealed with Parafilm, were kept in the growth chamber. After 10 days from the initiation of the experiment, the percentage of germinated spores was determined, and after 21 days, the differentiation stage of the gametophytes was observed. Spores were considered to have germinated if the rhizoid cell could be identified microscopically. For each variant, from which spores were randomly chosen, three replicates were performed for microscopic spore preparations to determine the germination percentage and the stage of gametophyte development. 

The state of the culture. For both experiments, culture dishes were kept in a POL EKO 350 growth chamber. The temperature values were 25 °C during daytime and 15 °C at night, and the humidity and light conditions were controlled (photoperiod: 16 h of light and 8 h of darkness).

### 2.2. Experiments on Animals

The biological materials used were Prussian carp samples (*Carassius gibelio*) and marsh frogs (*Pelophylax ridibundus*), captured from the surrounding lakes of Piteşti. 

#### 2.2.1. Experiments on Fish

The experiments on fish were carried out with the approval of the Bioethics Commission No. 13925 and the international guidelines of the European Parliament and the Council on the protection of animals used for scientific purposes according to Directive 2010/63/EU [33].

The experiments were carried out on male and female Prussian carps (*Carassius gibelio*) with an average weight of 14.54 ± 1.24 g, caught from the Argeș River, the Mihăilești downstream sector upstream from the Dâmbovița confluence. According to the most recent report—2020 [34]—on the state of water quality prepared by the Romanian National Water Administration, related to the Argeș–Vedea Basin Administration, the body of water is in poor chemical condition, with the substances that determined the non-attainment of the quality objective being: the insecticide heptachlor and heptachlor epoxide for the biota investigation environment. 

After 10 days of adaptation in the lab, where they were fed ad libitum once a day, the fish were divided into three groups of 10 fish each: the control group, without the addition of insecticide, and two experimental groups exposed to the Actellic 50 EC insecticide at concentrations of 0.002 µL L^−1^ and 0.004 µL L^−1^, corresponding to a concentration of 0.001 mg L^−1^ and 0.002 mg L^−1^ of pirimiphos-methyl, respectively. The tested concentrations were established on the basis of specialist work and preliminary tests (survival in the case of fish). Some authors have reported residues of pirimiphos-methyl in surface waters: 8.4–12.3 µg L^−1^ in runoff water from clay soil plots cultivated with potatoes and of differing soil surfaces [35]; 23.3 ppb in the New Damietta drainage canal (Egypt) [36]; and 0.38–3.03 ng L^−1^ in the river water dissolved phase (WDP), n.d., 1.54 ng L^−1^ in suspended particulate matter (SPM), and 2.23 ± 0.27 ng L^−1^ in sediment samples from the Sele River estuary, Southern Italy [37].

The exposure method chosen was the semi-static test; the immersion solution was changed every 24 h by transferring the fish to another aquarium, and the water was continuously aired. Fish were kept in 100 L glass aquaria with gently aerated tap water at a dissolved oxygen level of 7.53 ± 0.32 mg L^−1^, a pH of 7.83 ± 0.65, a total hardness of 100 mg L^−1^ CaCO3, and a natural light/dark photoperiod. The fish were not fed during the 14 days of the experiment in order to avoid the intervention of this factor [38]. 

In the three batches, we evaluated fish respiration by determining the oxygen consumption (closed breathing chamber method, using the Winkler method) and the respiratory rate of the fish at intervals of 24, 48, 72, 96, and 336 hours from the initiation of the experiments [38].

After 14 days of setting up the experiments, the fish were sacrificed, and blood samples were taken from the tail vein to determine the number of figure elements and the blood glucose value. The counting of figure elements was carried out under an Olympus microscope using Bürker counting chambers after prior staining with neutral red and crystal violet [38]. The glucose determination was performed from blood drops with a glucometer.

#### 2.2.2. Experiments on Marsh Frogs

The collection and handling of *Pelophylax ridibundus* eggs and tadpoles were carried out with the approval of the Bioethics Commission No. 13925 and in accordance with the provisions of Directive 2010/63/EU of the European Parliament and of the Council on 22 September 2010 on the protection of animals used for scientific purposes [33].

The tadpoles came from 3 egg masses collected from Strand Lake, bordering Pitești, in April 2022 and transported in isothermal packaging to avoid thermal and mechanical shocks. 

The eggs were distributed into 40L aquaria and maintained under controlled climatic conditions (temperature: 22 ± 0.5 °C; humidity: 70%; pH: 6.8 ± 0.14; photoperiod: 12 h light/12 h dark).

Two experimental variants were set up, in which we followed the action of Actellic 50 EC on the survival, growth, and development of the embryos and tadpoles of *Pelophylax ridibundus*. 

In variant I, we used eggs of *Pelophylax ridibundus*, randomly selected from the 3 egg masses collected, as follows:

- Control I, consisting of 30 eggs kept in dechlorinated, permanently aerated water under controlled climatic conditions for 9 days.
- Lot I.1, consisting of 30 eggs kept in a 0.1 mg L^−1^ Actellic 50 EC solution for 9 days.
- Lot I.2, consisting of 30 eggs kept in a 0.01 mg L^−1^ Actellic 50 EC solution for 9 days.
- Lot I.3, consisting of 30 eggs held in a 0.001 mg L^−1^ Actellic 50 EC solution for 9 days.

In all variants, the aquarium water was renewed every other day, and fresh working solutions were added to maintain the quality of the working solutions [39]. The number of hatched tadpoles was recorded daily, with dead individuals removed.

In variant II, we used tadpoles of *Pelophylax ridibundus*, obtained from the hatching of the rest of the eggs collected under the climatic conditions mentioned. After hatching, the tadpoles in Gosner stage 25 [40], the most common stage used in ecotoxicological research [41], were allocated to the following lots:

- Control II, consisting of 15 tadpoles kept in dechlorinated, permanently aerated and climate-controlled water for 5 days.
- Lot II.1, consisting of 15 tadpoles kept in a 0.1 mg L^−1^ Actellic 50 EC solution for 5 days.
- Lot II.2, consisting of 15 tadpoles in a 0.01 mg L^−1^ Actellic 50 EC solution for 5 days.
- Lot II.3, consisting of 15 tadpoles in a 0.001 mg L^−1^ Actellic 50 EC solution for 5 days.

The specimens used in the experiments were healthy, with their health status being determined by morphological appearance and the behavior of the individuals [42].

Throughout the experiment, the tadpoles were fed ad libitum with boiled and minced salad (boiled in boiling water for 4 min) [39].

The Actellic 50 EC concentration was verified by high-performance liquid chromatography (HPLC) using a Varian Pro Star chromatograph (Column: Agilent Poroshell 120 EC C18, mobile phase sol A: formic acid solution and B: acetonitrile). The measurements were performed with a Varian Liberty 110 spectrometer using a five-point calibration curve [43]. For the administration of the toxic substance, stock solutions were prepared and analytically checked daily. 

At the end of the experiment, biometric assessments of body length and volume were made, avoiding trauma as much as possible [44]. 

#### 2.2.3. Statistical Interpretation 

For the *Triticum* and fern test results, the data obtained were statistically analyzed using IBM SPSS Statistics 23 software. Means and standard errors were calculated, and the means were compared with Duncan’s test. The results obtained from Duncan’s test are shown in charts with letters (a, b, c, d, etc.) (*p* = 0.05). 

For the fish and tadpole tests, a statistical interpretation of the results obtained was performed using the ANOVA “one-way” test for all the physiological parameters investigated, comparing the results obtained in the groups exposed to the insecticide with those of the control group and the dynamic values of respiratory parameters from one determination interval to another. The results were judged to be significantly different using a significance threshold of *p* < 0.05. 

## 3. Results and Discussion

### 3.1. Phytotoxkit Microbiotest Application Results 

Germination was totally inhibited in the two dicotyledonous species *Lepidium sativum* and *Sinapis alba* for all the Actellic 50 EC concentrations tested. In the case of the monocotyledonous species *S. saccharatum*, the germination was inhibited by 25% compared to the control group for the higher concentrations of A0.1 and A0.01 of the insecticide. 

**Figure 1:**
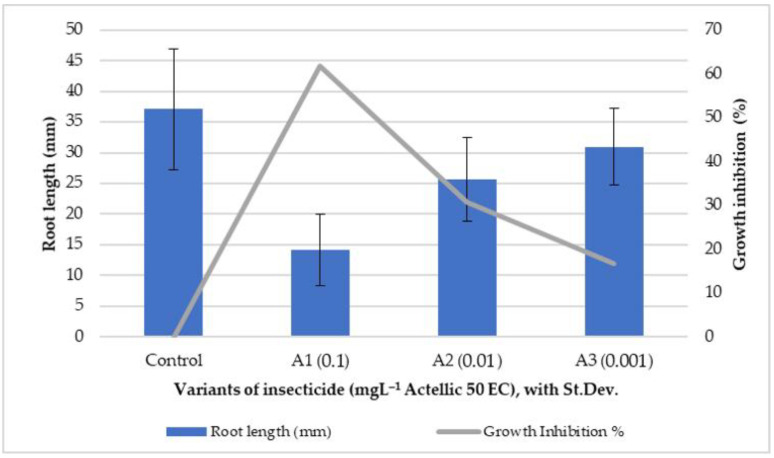
Root growth inhibition of *Sorghum saccharatum* after 3 days of exposure.

After 3 days of exposure, the root growth inhibition was recorded for sorghum and was found to be proportional to the increase in the Actellic 50 EC concentration in the soil (Figure 1).

The two dicotyledonous species (*Lepidium sativum* and *Sinapis alba*) showed a higher sensitivity (germination was totally inhibited) than *Sorghum saccharatum* (monocotyledonous), wherein germination was achieved at all tested concentrations. As for sorghum root growth, a percentage of 62% inhibition for the highest concentration of 0.1 mg L^−1^ was recorded. Similar results were obtained by applying a Phytotoxkit in the study to assess the risk of infiltration into soil/groundwater of long-term buried/stored insecticides among the three species used, with *Lepidium* and *Sinapis* demonstrating a greater sensitivity than sorghum [21]. In another study, the growth index values revealed the following increasing order of plant sensitivity to contaminated sediments: *L. sativum* < *S. alba* < *S. saccharatum* [45,46].

**Figure 2:**
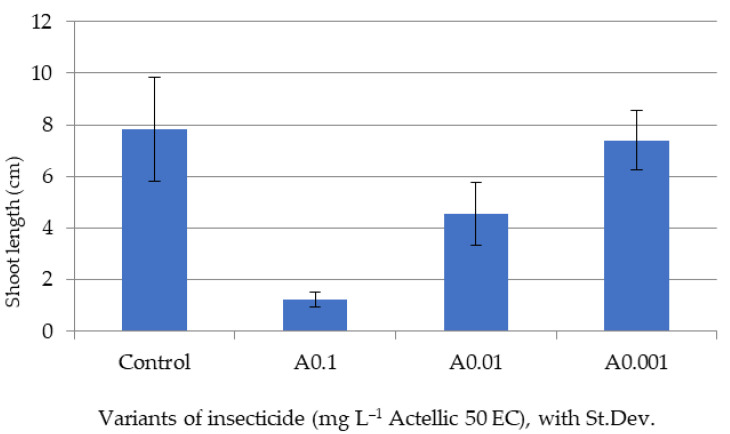
Average shoot length of sorghum plants after 5 days of exposure.

**Figure 3:**
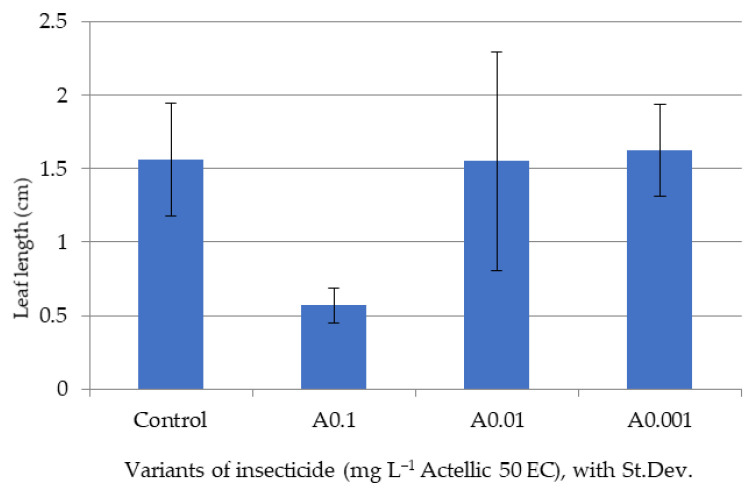
Average leaf length of sorghum after 5 days of exposure.

**Figure 4:**
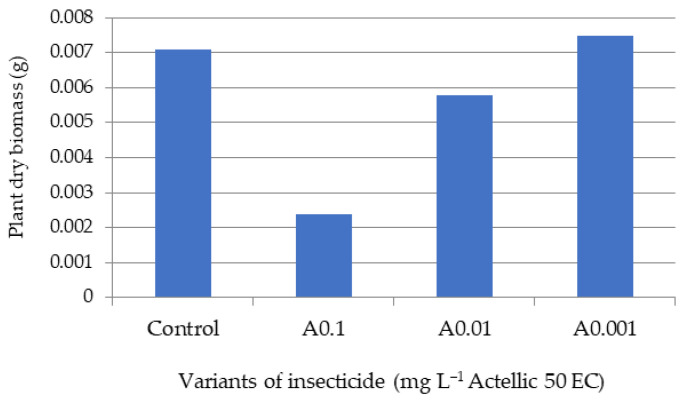
Average dry biomass of sorghum after 5 days of exposure.

**Figure 5:**
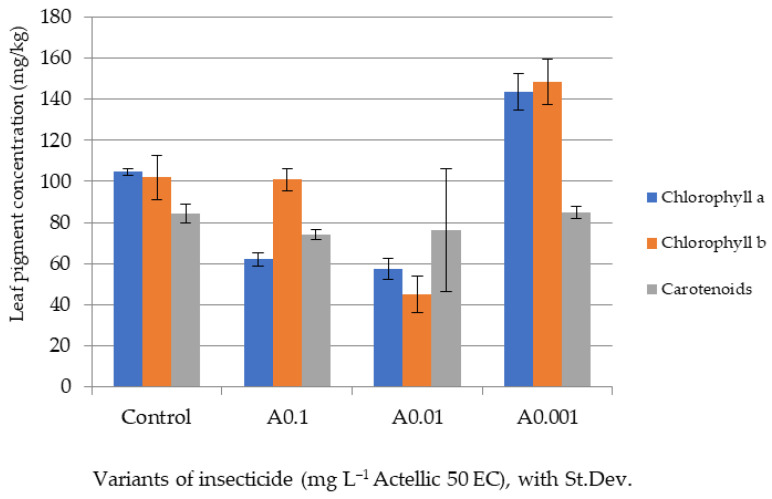
Average concentration of foliar pigments in sorghum plants after 5 days of exposure.

Only after five days of exposure, once the stem and leaves had sufficiently developed, were we able to make biomass and pigment determinations on the tested lots (Figure 2, Figure 3, Figure 4 and Figure 5). 

The biometric data showed a high variability among plants of the same batch. However, the minimal values of shoot and leaf growth and of final biomass were all reported for the A0.1 experimental variant. The A0.001 batch had values of aerial organ lengths and a biomass that were most similar to those of the control (Figure 2, Figure 3 and Figure 4).

Figure 5 shows the average concentrations of foliar pigments. The control plants had over 100 mgkg^−1^ of each chlorophyll type and 84 mgkg^−1^ of total carotenoids. While the concentrations of carotenoids remained similar in all the experimental batches (74–85 mgkg^−1^), there were high variations in the chlorophyll content. Variants A0.1 and A0.01 showed a decrease in chlorophyll concentration (especially chlorophyll *a*), and in variant A0.001, an increase was observed in the concentration of both chlorophylls.

The organophosphorus insecticides tested in the present study are known to have various physiological effects on plant growth. A study involving several such insecticides sprayed on cabbage plants showed that organophosphates affect the normal synthesis of chlorophylls (both *a* and *b*, either through an enhanced breakdown or a lower synthesis rate). After each spray, the total chlorophyll content can drop by 15–20%. However, while pirimiphos-methyl was found to cause a major initial drop, compared to other similar pesticides (malathion, prothiophos), the recovery of foliar pigment levels was also more pronounced with this compound [47].

A chlorophyll reduction (along with a reduction in other compounds, such as sugars, proteins, and RNA) was also observed in wheat plants after treatment with organophosphates (pirimicarb, dimethoate) [48].

Tomatoes and cucumbers are other species whose foliar pigment synthesis can be affected by organophosphate spraying (dimethoate, profenofos). The response depends on the pigment type and species. For instance, profenofos caused a higher decrease in chlorophyll *b* levels in tomato and chlorophyll *a* in cucumber plants, while the effects on carotenoids were mixed, with an initial drop followed by a major increase [49].

Besides leaves, abnormal pigment levels have also been observed in fruits. In green peppers, Actellic 50 EC treatments induced a 38% increase in the β-carotene concentration [50].

As for plant growth parameters, a field study on onion plants (pirimiphos-methyl in a regular dosage of 0.5%) found significant increases in the average plant height, number of leaves, fresh and dry biomass (up to 100%), bulb yield, etc. However, such effects in field experiments were probably indirect, due to lowering the population of herbivorous insects [51]. Similar data were found for okra plants (*Abelmoschus esculentus*) treated with Attack (another insecticide containing pirimiphos-methyl), with some increases in root weight (10%) and shoot weight (16%), plus other parameters [52].

### 3.2. Influence of Actellic 50 EC on - Growth of Triticum aestivum Seedlings

**Figure 6:**
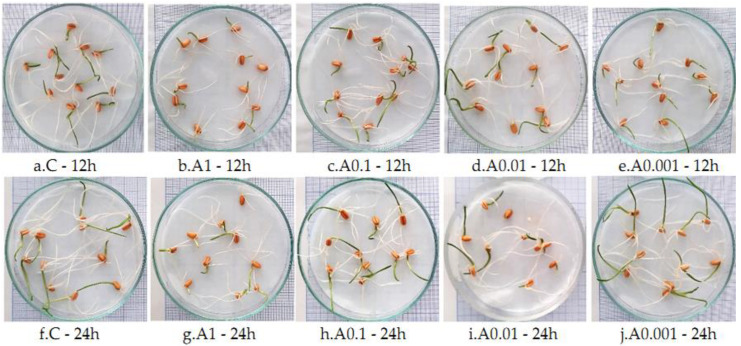
(**a**–**e**) *Triticum aestivum* seedlings 5 days after exposure to Actellic 50 EC for 12 h; (**f**–**j**) *Triticum aestivum* seedlings 5 days after exposure to Actellic 50 EC for 24 h.

Actellic 50 EC exposure significantly inhibited the root and stem growth in *T. aestivum* (Table 2). The inhibition varied with concentration and time. The shortest root and stem lengths were observed in the A1 variants, regardless of exposure time (Table 2, Figure 6). For this reason, A1 also had a lower value for fresh weight (Table 2). The 24 h exposure to Actellic 50 EC inhibited root growth more than the 12 h exposure. The sensitivities recorded for the parameters of interest were as follows: root > stem > fresh weight > dry weight.

Actellic (pirimiphos-methyl) induces various physiological and biochemical changes in plants, depending on the species, the dose applied, the ontogenetic stage of the plant, and other factors. Kandil et al. [53] studied the effect of sprayed Actellic on the germination and seedling parameters of wheat (*Triticum aestivum* cultivar Misr 1). They observed that Actellic (375 mL/100 L) significantly decreased the percentage of abnormal seedlings and increased the final germination percentage, germination rate index, germination energy, root and shoot length, seedling dry weight, and seedling vigor index. The effect of Actellic on two-month-old cabbage plants was studied by Shams-El-Din et al. [47]. The chlorophyll content decreased after the second and third treatments. A similar situation was observed for the sugar and carbohydrate content, as well as for protein content. According to the elemental analysis, the N content decreased while the P content increased; for the K content, no fixed trend was observed. Sharma et al. [54] confirmed that pesticide applications negatively affect plant growth and development by reducing chlorophyll and protein content and by decreasing photosynthetic efficiency. 

### 3.3. Influence of Actellic 50 EC on Fern Spore Germination and Gametophyte Differentiation

**Figure 7:**
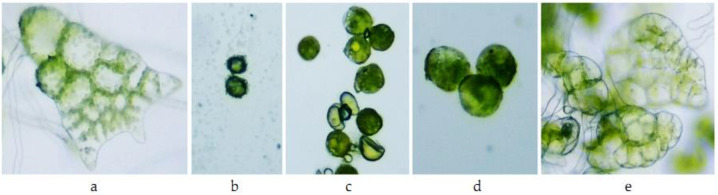
*Athyrium filix-femina* gametophyte after 21 days of continuous exposure to Actellic 50 EC: (**a**) C (100×)—prothallium blade; (**b**) A1 (100×)—ungerminated spores; (**c**) A0.1 (100×)—ungerminated spores, slightly swollen; (**d**) A0.01 (100×)—germinated spores; and (**e**) A0.001 (100×)—prothallium blade.

**Figure 8:**
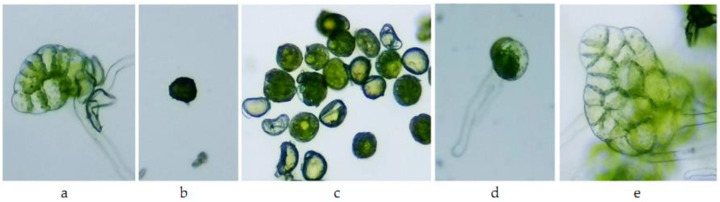
*Asplenium scolopendrium* gametophyte after 21 days of continuous exposure to Actellic 50 EC: (**a**) C (100×)—prothallium blade; (**b**) A1 (100×)—ungerminated spores; **(c)** A0.1 (100×)—ungerminated spores, slightly swollen; (**d**) A0.01 (100×)—germinated spores; and **(e)** A0.001 (100×)—prothallium blade.

For fern spores, the situation was more dramatic than for *Triticum aestivum*: in variant A1 of both species, the spores did not germinate (Table 3). In variants A0.1 and A0.01, the percentage of germinated spores was less than 10% and 20%, respectively. Even though, for variant A0.001, the percentage of germinated spores recorded was 60% for *A. scolopendrium* and 74% for *Athyrium filix-femina*, the prothallium blades that were observed after 21 days were discolored (Table 4, Figure 7 and Figure 8).

Morphological, physiological, and biochemical changes under the influence of different types of pesticides have been reported in a range of ferns in many non-target species [55]. The discoloration observed in prothallial blades obtained under the influence of 0.001 mg L^−1^ of Actellic 50 EC indicated deterioration of chlorophyll pigments in the two species tested, *Asplenium scolopendrium* and *Athyrium filix-femina*. In general, insecticides significantly influence spore germination, cause delays in the formation of characteristic gametophyte stages and the necrosis of prothallial cells, and affect the elongation of gametophyte rhizoids [56].

### 3.4. The Action of Actellic 50 EC on Fish

#### 3.4.1. Influence of Actellic 50 EC on Survival 

During the acute test (96 h), we recorded mortality in both groups exposed to Actellic 50 EC (10% in group I and 30% in group II). At the end of the 14-day experiment, the survival percentage in group I was 70%, and in group II, it was 60%. Fish were considered dead when they showed no opercular movements and no reaction to touch with a glass rod.

Omoregie and Ufodike [57] studied the toxicity of the insecticide Actellic 25 EC in the species *Oreochromis niloticus* using the continuous flow test at 21.34 ± 0.39 °C. At the end of the acute test (96 h), the mortality levels recorded were 30, 40, 60, and 85% at the test concentrations of 1.563, 3.125, 6.25, and 12.5 µg L^−1^ of Actellic 25 EC, respectively. 

#### 3.4.2. Influence of Actellic 50 EC on Behavior

Behavioral changes are the most sensitive indicators of potential toxic effects, with most insecticides altering behavioral patterns by interfering with the nervous system and sensory receptors [58]. The inhibition of acetylcholine esterase activity is considered a specific biomarker of organophosphorus insecticide exposure [59].

If the heptachlor insecticide (mentioned in the monitoring water quality report [34]) is present in the water from which the fish come, even in low quantities, but for a long term, it can influence the fish. The organochlorine insecticide heptachlor and its metabolite, heptachlor epoxide, can induce metabolic changes in fish by blocking GABA receptors, which results in an overstimulation of the nervous system [60].

Forty-eight hours after exposure to the Actellic 50 EC insecticide, the carps of both groups showed a number of behavioral changes: restlessness; hyperactivity followed by increasingly weak swimming; a loss of balance; and, especially at 96 h after exposure, increased mucus secretion. Similar symptoms of Actellic 25 EC intoxication were also observed by Lawal and Samuel [9] in representatives of the species *Poecilia reticulata* during the acute test. 

The reversibility of the behavioral effects of organophosphorus pesticides (due to the long-term inhibition of brain acetylcholinesterase) has been reported in *Carassius auratus* [61] and *Tilapia oreochromis mossambicus* [62].

In a 3-month chronic toxicity study on the sensitivity of Atlantic salmon to the insecticide Actellic 50 EC present in plant-based feeds, Berntssen et al. [63] reported a reduction in acetylcholinesterase activity, which is the basis of behavioral changes.

#### 3.4.3. Influence of Actellic 50 EC on Respiratory Rate and Average Oxygen Consumption

Variations in the respiration rate and oxygen consumption of carps exposed to Actellic 50 EC insecticide at two concentrations (0.002 and 0.004 µL L^−1^) are shown in Table 5 and Table 6.

The respiratory rate of carps exposed to Actellic 50 EC decreased significantly from the first 24 h after the fish were introduced to the insecticide solutions, with the mean values recorded at the end of the acute test (after 96 hours of exposure) being 8.83% lower than the initial values at 0.002 µL L^−1^ and 26.3% lower at 0.004 µL L^−1^. The values of this physiological parameter determined at the end of the experiments showed decreases compared to the values determined before the exposure of the carps to the insecticide (by 21.21% at the concentration of 0.002 µL L^−1^ and 36.24% at the concentration of 0.004 µL L^−1^).

Decreased opercular ventilation in response to exposure to 0.002 mg L^−1^ was also reported by Omoregie and Ufodike [57] in *Oreochromis niloticus niloticus*, with the authors observing a significant decrease in the frequency of opercular movements after the first 24 h after exposure to the insecticide Actellic 25 EC, followed by a less marked decrease in this physiological index until the end of the acute test. 

The decrease in oxygen consumption can be linked to the effect of the insecticide at the gill level, as organophosphorus insecticides affect the respiratory organs of fish (temephos and deltamethrin affect the gills of *Aphanius disparus* species), chloral cell degradation, shedding, detachment, epithelial lifting, hypertrophy, and gill fusion [64,65].

A decreased oxygen consumption due to organophosphorus insecticide exposure has also been reported [66] for *Ctenopharyngodon idella* in nuvan poisoning under static experimental conditions.

The second respiratory parameter determined, the mean oxygen consumption, also decreased significantly in fish poisoned with Actellic 50 EC, with the values recorded after 14 days of exposure to the pesticide being 24.8% lower than the initial values at 0.001 mg L^−1^ and 26.06% lower at 0.002 mg L^−1^.

Similar results were recorded for Dichclorvos, another organophosphate insecticide, in a 96-hour exposure: a decreased frequency of opercular movements in *Channa punctatus* [67]. 

In addition, the results of sub-chronic exposure could be compared with similar results reported by other authors for Dichlorvos: a reduced respiratory rate in *Heteropneustes fossilis* after exposure for 30 days at 0.44 mg L^−1^ [68], and in *Tilapia mossambica* after exposure for 21 days at 0.5–1 mg L^−1^ [69]. 

#### 3.4.4. Influence of Actellic 50 EC on Hematological Parameters: Blood Figure Elements and Glucose 

Pesticides are an important hematological stressor for aquatic fauna, especially fish, but there is no single pattern of hematological changes for all classes of pesticides [70]. Blood figure counts are considered a bioindicator of fish poisoning following exposure to organophosphorus insecticides [70].

The mean red and white blood cell counts were significantly increased in carps exposed to Actellic 50 EC for 14 days at both concentrations investigated (Table 7).

Mgbenka et al. [6] reported adverse effects of the Actellic insecticide: leukocytosis and significantly decreased hemoglobin, hematocrit, and mean erythrocyte counts in *Clarias albopunctatus* as a result of 18 days of exposure to the insecticide (0.3, 0.5, 0.8, and 1.0 mg L^−1^) at 27 °C. 

In another study on Actellic, Oluah and Mgbenka [71] reported increased leukocyte counts for the same exposure period and the same species at concentrations of 0.3, 0.5, 0.8, and 1.0 µg L^−1^.

Nannu et al. [72] reported increased mean white blood cell counts after the exposure of *Oreochromis niloticus* to Kinalux 25 EC (an organophosphorus insecticide). 

The mean glycemia values were significantly higher after 14 days of exposure to the Actellic 50 EC insecticide (15.62% higher in carps exposed to 0.001 mg L^−1^ of insecticide compared to the control group and 28.65% higher in those exposed to 0.002 mg L^−1^). 

Similar results of an increase in blood glucose as a result of Actellic exposure were reported by Olsvik et al. [73]. The authors reported on the effect of the insecticide pirimiphos-methyl on glycolysis from an in vitro study on *Salmo salar* hepatocytes (glycolytic activity in hepatocytes decreased, either due to decreased enzyme activity or decreased uptake of glucose from the blood). Increased blood glucose in Dichlorvos intoxication was reported by Gautam et al. [74] for *Clarias batrachus* and by Srinivas et al. [75] for *Catla catla*. Significant increases in blood glucose have been reported in different species when exposed to Fenitrothion: *Oreochromis niloticus* [76], *Sarotherodon mossambicus* [77], and *Heteropneustes fossilis* [78]. Glycogen breakdown at the liver and muscle levels has been reported in several studies on fish exposed to organophosphorus insecticides: in Clarias batrachus following exposure to dimethoate and Rogor [79,80], and in *Oreochromis mossambicus* following exposure to RPR-II (2-butenoic acid-3-(diethoxyphosphinothioyl) methyl ester) [81].

Changes in hematological parameters can result in alterations to the vital physiological processes of fish: respiration, feeding, reproduction, etc. These parameters could thus serve as sensitive indices to examine the health status and to ascertain the toxic effects on ecosystems under pesticide exposure, as they can provide early warning signals of pesticide exposure, and toxicological effects can be followed by monitoring [70,82,83].

### 3.5. Results for Marsh Frogs

The exposure of *Pelophylax ridibundus* eggs to different concentrations of pirimiphos-methyl showed a negative influence on the hatching percentage (Table 8). Thus, at a concentration of 0.1 mg L^−1^ of Actellic 50 EC, there was a delay in hatching, but also a reduction in the number of hatched embryos (66.6%). Less toxic was the concentration of 0.001 mg L^−1^ of Actellic 50 EC, with a hatching percentage of 82.58%. 

Delayed metamorphosis or a reduced body size may be associated with a lower survival rate, which may have unfavorable consequences for the species [84]. 

The survival of *Pelophylax ridibundus* tadpoles during the sub-acute test period at all three concentrations investigated is shown in Table 9.

An analysis of the data showed that the least toxic concentration was 0.001 mg L^−1^ of Actellic 50 EC, with a survival rate of 100% at the end of the subacute test, while a concentration of 0.1 L^−1^ of Actellic 50 EC resulted in a survival rate of 50%.

Analyzing the influence of the pesticide on body length and volume, a decrease in the values of these parameters was observed in specimens intoxicated with Actellic 50 EC at a concentration of 0.1 mg L^−1^ in water, while a concentration of 0.001 mg L^−1^ of Actellic 50 EC did not cause significant changes at the end of the subacute test (Table 10).

Similar results were observed by Lanctôt et al. [85] in studies on the survival, growth, and development of wood frog (*Lithobates sylvaticus*) tadpoles under glyphosate action, indicating a decrease in body length. Moreover, pendimethalin administered in sublethal doses caused biochemical changes in the antioxidant enzymes of zebrafish larvae [86]. 

Exposure to different doses of dinitramine resulted in malformations in zebrafish embryos and the onset of cardiotoxicity [87]. The agricultural use of chlorpyrifos poses a risk to amphibian species, as it contributes to the immunosuppression of tadpoles and decreases their survival rate [88]. 

### 3.6. Evaluation of the Effects of Organophosphorus Insecticides on Genotoxicity and Reproductive Function, Related to the Period of Exposure (Acute, Sub-Chronic, and Chronic Toxicity)

Studies on sub-chronic and chronic toxicity assessments and the impact on reproduction and genotoxicity were conducted in laboratory animals (mice and rats), targeting the risk of chronic exposure of animals or humans in areas of the improper storage and handling of pesticides in the agricultural sector and related activities. 

Related to the chronic impact on genotoxicity and reproductive function, in a chronic exposure of rats (45 days) to phosalone (PLN), Amniattalab and Razi [89] noted adverse effects on testicular tissue. PLN reduced the potential for fertilization and the development of embryos, acting on the testicles and sperm; it promoted its impact by increasing DNA and RNA damage and by decreasing testicular endocrine activity and antioxidant status.

Regarding the impact on genotoxicity, Khodabandeh et al. [90] studied the effects of Zolone (phosalone) in a 5-day exposure on mice bone marrow-derived cells, observing a time and dose-dependent toxicity that caused further DNA degradation; one of the most important mechanisms of toxicity was an increase in the oxidative reactions of tissues due to a decrease in the effectiveness of the enzymes involved in antioxidant defense, ultimately accelerating cell aging and possibly leading to genetic damage of the cells [90].

As pointed out by Khodabandeh et al. [90], the risks of chronic exposure to these compounds are due to their residues in food or because of poor working conditions, a lack of personal protective equipment, and inadequate training on the dangers of construction and exposure to agricultural land. Chronic exposure leads to disorders of the immune system, neurological diseases, endocrine disorders, miscarriage, degenerative diseases, and cancers.

Unfortunately, there are not enough data on the levels of concentration and types of organophosphorus insecticides existing in the water basins, as well as the quantities applied to the agricultural land, which would be necessary so that the level of risk for the different non-target organisms could be assessed. A monitoring program would be absolutely necessary. These data would be useful precisely to be able to assess the intensity and duration of exposure to these toxic compounds of non-target organisms, in order to initiate studies on the chronic effects on the exposed organisms.

## 4. Conclusions 

The insecticide Actellic 50 EC adversely affected the germination of the seeds and spores of the non-target species tested, producing either complete inhibition of germination of some of the tested species or a decrease in the percentage of germination. The sub-chronic and chronic phytotoxicity generated by exposure to Actellic 50 EC manifested by affecting the growth process of seedlings, which could ultimately lead to important changes in the life cycle, reproduction, and productivity. The continuous exposure of *A. scolopendrium* and *A. filix-femina* spores to Actellic 50 EC induced significant changes in germination and gametophyte differentiation, suggesting impaired in situ reproduction in contaminated ecosystems. 

Intoxication with Actellic 50 EC manifests itself in Prussian carp by causing changes in respiratory function, an increase in the average number of white blood cells and plasma glucose level (as a result of the reduction in hepatic glycolysis), and a series of behavioral changes (due in particular to the decrease in the activity of cerebral acetylcholinesterase), which can affect fish feeding and survival. The administration of the Actellic 50 EC insecticide at concentrations of 0.1 mg L^−1^, 0.01 mg L^−1^, and 0.001 mg L^−1^ negatively influenced the hatching rate of *Pelophylax ridibundus* embryos as well as the survival, body length, and volume of tadpoles, which are changes that affect their survival as adults. 

After highlighting the acute and sub-chronic toxicity of the organophosphorus insecticide Actellic 50 EC on the non-target species tested, we recommend continuing to investigate other parameters in order to complete the profile of changes induced by the exposure of different groups of organisms to this xenobiotic. It is important that these tests also include various plant species, which constitute the first trophic level in most ecosystems. 

Given the high degree of toxicity of the insecticide Actellic 50 EC, we recommend continuing investigations on non-target species, plants, and animals, as the sub-chronic and chronic effects are quite little known in the scientific literature.

Our study can thus contribute to the application of better measures to monitor and protect ecosystems against the uncontrolled spread of the tested pesticide. 

## Figures and Tables

**Table 1:** The experimental variants.

	*Triticum aestivum* Seeds	Fern Spores
Control	Distilled water	Knop solution
A1	1 mg L^−1^ of Actellic 50 EC	1 mg L^−1^ of Actellic 50 EC
A0.1	0.1 mg L^−1^ of Actellic 50 EC	0.1 mg L^−1^ of Actellic 50 EC
A0.01	0.01 mg L^−1^ of Actellic 50 EC	0.01 mg L^−1^ of Actellic 50 EC
A0.001	0.001 mg L^−1^ of Actellic 50 EC	0.001 mg L^−1^ of Actellic 50 EC

Concentrations express the amount of the insecticide’s active substance.

**Table 2:** Influence of Actellic 50 EC on *Triticum aestivum* seedling growth.

Variant	Root		Stem		Fresh Weight		Dry Weight	
	12 h	24 h	12 h	24 h	12 h	24 h	12 h	24 h
Control	27.43 ± 3.31 ^a^	34.27 ± 4.10 ^a^	31.67 ± 1.87 ^a^	35.47 ± 2.14 ^a^	1.02 ± 0.04 ^ab^	1.20 ± 0.07 ^a^	0.35 ± 0.02 ^b^	0.38 ± 0.01 ^a^
A1	16.53 ± 2.03 ^b^	10.40 ± 1.51 ^b^	16.50 ± 1.37 ^c^	13.70 ± 1.44 ^c^	0.90 ± 0.05 ^b^	0.85 ± 0.08 ^b^	0.35 ± 0.02 ^ab^	0.39 ± 0.02 ^a^
A0.1	23.27 ± 1.45 ^ab^	17.00 ± 1.69 ^b^	23.83 ± 1.27 ^b^	25.13 ± 1.30 ^b^	1.08 ± 0.06 ^a^	0.98 ± 0.06 ^ab^	0.40 ± 0.02 ^a^	0.37 ± 0.01 ^a^
A0.01	21.87 ± 1.98 ^ab^	14.07 ± 2.53 ^b^	24.33 ± 2.00 ^b^	23.17 ± 2.20 ^b^	1.00 ± 0.06 ^ab^	0.96 ± 0.12 ^ab^	0.37 ± 0.00 ^ab^	0.38 ± 0.01 ^a^
A0.001	16.90 ± 2.38 ^b^	16.00 ± 2.33 ^b^	23.77 ± 1.74 ^b^	28.07 ±2.00 ^b^	0.93 ± 0.02 ^ab^	1.01 ± 0.07 ^ab^	0.36 ± 0.02 ^ab^	0.40 ± 0.02 ^a^
F	3.91	12.65	10.27	18.22	2.07	2.24	2.03	0.69

a,b: Interpretation of the significance of differences using Duncan’s test, *p* = 0.05.

**Table 3:** Influence of Actellic 50 EC on spore germination.

Germination Percentage (%)		
Variants	*Athyrium* *filix-femina*	*Asplenium scolopendrium*
Control	81.33 ± 1.86 ^a^	75.33 ± 2.33 ^a^
A1	0.00	0.00
A0.1	6.67 ± 0.67 ^d^	1.00 ± 0.58 ^d^
A0.01	18.00 ± 2.65 ^c^	13.33 ± 1.86 ^c^
A0.001	74.00 ± 1.53 ^b^	60.00 ± 1.73 ^b^
F	565.29	508.69

a–d: Interpretation of the significance of differences using Duncan’s test, *p* = 0.05.

**Table 4:** Influence of Actellic 50 EC on gametophyte differentiation.

Variants	*Athyrium filix-femina*	*Asplenium scolopendrium*
M	prothallium blade	filament and prothallium blade
A1	ungerminated spores	ungerminated spores
A0.1	ungerminated spores, slightly swollen	ungerminated spores, slightly swollen
A0.01	germinated spores	germinated spores
A0.001	prothallium blade slightly discolored	prothallium blade discolored

**Table 5:** Variations in the average respiratory rate (breaths/minute) and the standard deviation of the Prussian carps exposed to Actellic 50 EC insecticide at different concentrations.

Lots	0 h	24 h	48 h	72 h	96 h	168 h	336 h
**Control**	84.2 ± 2.5	82.7 ± 4.22	78.86 ± 4.24	80.6 ± 2.74	83.5 ± 4.56	82.4 ± 3.42	81.8 ± 6.58
Lot I (Actellic 50EC 0.001 mg L^−1^)	81.6 ± 3.48	76.7 ± 4.38 *	74.5 ± 6.48	72.78 ± 9.82 *	74.4 ± 5.26	68.3 ± 4.86 *	64.3 ± 2.48 *
Lot II (Actellic 50 EC 0.002 mg L^−1^	88.6 ± 7.22	72.6 ± 6.25 *	65.5 ± 5.48 *	62.8 ± 6.14	65.3 ± 3.92	61.3 ± 4.16 *	56.5 ± 5.48 *

* The mean difference was significant at the 0.05 level.

**Table 6:** Variations in the average oxygen consumption (mL of oxygen/kilogram/hour) and the standard deviation of the Prussian carp exposed to Actellic 50 EC insecticide at different concentrations.

Lots	0 h	24 h	48 h	72 h	96 h	168 h	336 h
**Control**	142 ± 11.36	146 ± 6.82	144 ± 5.56	148 ± 4.86	146 ± 8.56	148 ± 5.74	138 ± 9.52
Lot I (Actellic 50EC 0.001 mg L^−1^)	148 ± 7.62	128.7 ± 6.38 *	122.1 ± 7.58*	113.78 ± 9.82 *	94.4 ± 7.56 *	98.3 ± 7.83	111.3 ± 8.65 *
Lot II (Actellic 50EC 0.002 mg L^−1^)	138.4 ± 11.14	107.8 ± 8.65 *	95.5 ± 7.56 *	82.4 ± 6.87 *	74.3 ± 3.94 *	78.3 ± 4.47	88.5 ± 9.84 *

* The mean difference was significant at the 0.05 level.

**Table 7:** Mean number of figure elements and glucose with standard deviations in carp exposed to Actellic 50 EC.

**Lots**	**Red Blood Cells/mL Blood**	**White Blood Cells/mL Blood**	**Glucose (mg/100 mL Blood)**
**Control lot**	958,625 ± 11,310	51,250 ± 5648	64.54 ± 9.28
**Lot I (Actellic 50 EC 0.001 mg L^−1^)**	1,106,500 ± 28,550 *	56,680 ± 6545 *	74.63 ± 6.68 *
**Lot II (Actellic 50 EC 0.002 mg L^−1^)**	1,118,600 ± 35,560 *	62,480 ± 4850 *	83.03 ± 7.42 *

* The mean difference was significant at the 0.05 level.

**Table 8:** The influence of Actellic 50 EC insecticide in different concentrations on the percentage of hatching success.

Actellic 50 EC Concentration (mg/L water)	Percentage of Hatching (Days)								
	1	2	3	4	5	6	7	8	9
Control	0	0	6.66	33.33	59.94	86.58	86.58	86.58	86.58
Lot I.1 (0.1)	0	0	0	6.66 *	26.64 *	33.33 *	56.61 *	63.27 *	66.6 *
Lot I.2 (0.01)	0	0	0	29.95 *	43.27 *	59.92 *	63.25 *	73.25 *	73.25 *
Lot I.3 (0.001)	0	0	3.33	36.63	59.94	73.25	73.25	82.58	82.58

* The mean difference was significant at the *p* = 0.05 level.

**Table 9:** The percentage of survival on *Pelophylax ridibundus* tadpoles intoxicated with Actellic 50 EC insecticide.

Variable	Experimental lots						
		Start Point	24 h	48 h	72 h	96 h	120 h
Percentage of survival	Control II 0.1 mg L^−1^Actellic 50 EC 0.01 mg L^−1^Actellic 50 EC 0.001 mg L^−1^Actellic 50 EC	100 100 100 100	100 70 * 80 * 100	100 70 * 80 * 100	100 60 * 80 * 100	100 60 * 70 * 100	100 50 * 70 * 100

* The mean difference was significant at the *p* = 0.05 level.

**Table 10:** Mean ± SD for volume and body length of *Pelophylax ridibundus* tadpoles upon Actellic 50 EC action.

Variable (Mean ± SD)	Control II	Lot II.1	Lot II.2	Lot II.3
Volume of the tadpoles (mL)	0.604 ± 0.020 ^a^	0.475 ± 0.029 ^d^	0.501 ± 0.015 ^c^	0.566 ± 0.018 ^b^
Body length of the tadpoles (cm)	3.74 ± 0.126 ^a^	3.1 ± 0.105 ^c^	3.4 ± 0.176 ^b^	3.66 ± 0.117 ^a^

a–d: Interpretation of the significance of differences using Duncan’s test, *p* = 0.05.

## Data Availability

Not applicable.
